# Single-Cell Profiling Reveals Heterogeneity of Primary and Lymph Node Metastatic Tumors and Immune Cell Populations and Discovers Important Prognostic Significance of CCDC43 in Oral Squamous Cell Carcinoma

**DOI:** 10.3389/fimmu.2022.843322

**Published:** 2022-03-24

**Authors:** Zhenyu Wang, Hongbo Zhang, Yanan Zhai, Fengtong Li, Xueying Shi, Muying Ying

**Affiliations:** ^1^ Department of Molecular Biology and Biochemistry, Basic Medical College of Nanchang University, Nanchang, China; ^2^ Medical College of Nanchang University, Nanchang, China

**Keywords:** single-cell analysis, tumor immune microenvironment, *CCDC43*, clinical prognosis, oral squamous cell carcinoma (OSCC)

## Abstract

Although substantial progress has been made in biological research and clinical treatment in recent years, the clinical prognosis of oral squamous cell carcinoma (OSCC) is still not satisfactory. Tumor immune microenvironment (TIME) is a potential target, which plays an essential role in the response of anti-tumor immunity and immunotherapy. In this study, we used scRNA-seq data, revealing the heterogeneity of TIME between metastatic and primary site. We found that in the metastatic site, the content of cytotoxic T cells and classical activated macrophages (M1 macrophages) increases significantly, while alternately activated macrophages (M2 macrophages) and inflammatory cancer-associated fibroblasts (iCAFs) decrease, which may be due to the increased immunogenicity of OSCC cells in the metastatic site and the changes in some signal pathways. We also found that iCAFs may recruit alternately activated macrophages (M2 macrophages) by secreting *CXCL12*. Then, we described a regulatory network for communication between various TIME cells centered on OSCC cells, which can help to clarify the possible mechanism of lymph node metastasis in OSCC cells. By performing pseudotime trajectory analysis, we found that the expression *CCDC43* is upregulated in more advanced OSCC cells and is an independent prognostic factor for poor living conditions. Other than this, the high expression of *CCDC43* may impair the antitumor immunity of the human body and promote the metastasis of OSCC cells. Our research provides a profound insight into the immunological study of OSCC and an essential resource for future drug discovery.

## Introduction

Oral cancer is currently the 11th most common cancer around the world, of which about 90% is oral squamous cell carcinoma (OSCC). More than 300,000 new cases of OSCC occur each year, and 145,000 people die as a result (1). The occurrence of OSCC is the result of the comprehensive action of complex genetic and environmental factors. Smoking, drinking, human papillomavirus infection, and betel nut chewing are all key causes of OSCC (2–5). The traditional treatment of OSCC mainly includes surgical resection, radiotherapy, and chemotherapy. Although great progress has been made in various treatments, the 5-year survival rate of OSCC is still only about 50% (6), and its recurrence rate is also at a high level (7).

In recent years, the immunotherapy of tumor has developed rapidly. For instance, antitumor chimeric antigen receptor T-cell immunotherapy shows great potential in tumor treatment (8). Application of immune checkpoint inhibitors represented by *PD-1*/*PD-L1* and *CTLA4* inhibitors has greatly improved the patients’ prognosis of various cancers (9). However, in the specific process of treatment, immune checkpoint inhibitors also expose many limitations. For example, this kind of treatment can only have therapeutic benefits for a small number of patients and has caused many immune-related adverse events (10, 11). The reasons for the above results are in addition to the different pathological features of tumor patients; the heterogeneity of tumor immune microenvironment (TIME) is also an essential factor that cannot be ignored (12, 13). TIME is defined as the cellular environment in which tumors exist, and its immune pattern can inhibit or promote the occurrence and development of tumors (13). The cell types in TIME include various immune cells (T cells, B cells, dendritic cells, macrophages, etc.), tumor-associated fibroblasts, and vascular endothelial cells. The TIME components of different types of tumors and different pathological stages of the same tumor have great heterogeneity, which plays a key role in tumor progression and metastasis (14). The TIME of head and neck squamous cell carcinoma (HNSCC) consists of many different cell subsets that infiltrate the tumor and interact with tumor cells or through various networks. Previous studies have shown that the high metastatic and recurrence rates of HNSCC may be caused by the interaction of tumor cells with the surrounding tissue matrix and immune cells that make up TIME (15). This interaction relationship may be an important factor affecting the immunotherapy of HNSCC patients. For instance, Kulasinghe et al. found that many immune cell types and protein markers (CD4, CD68, etc.) in TIME have important potential in predicting the progress of HNSCC and the effect of immunotherapy (16). As an important type of HNSCC, elucidating the differences of components in different stages, cell-to-cell interactions, and the heterogeneity of TIME will be helpful to further study the occurrence and development of OSCC and improve the effect of immunotherapy.

In the development in recent years, single-cell RNA-sequencing (scRNA-seq) shows its unique advantages in the study of heterogeneous gene expression in different tissue samples (17). In the present study, through the comprehensive use of scRNA-seq data and bulk RNA-seq data, we studied the diversity of tumor immune microenvironment between the primary site of OSCC and the site of lymph node metastasis and found that there is significant heterogeneity not only in OSCC tumor cells but also in immune cells. We also found that there are significant differences in the mode of communication between cells that make up the components of TIME between the primary and metastatic sites. In addition, the expression of *CCDC43* is found to be significantly associated with the clinical prognosis and antitumor immunity of OSCC, which may be an important potential biomarker.

## Materials and Methods

### Data Acquisition and Preprocessing

The scRNA-seq data of OSCC samples (accession number GSE103322) were downloaded from the Gene Expression Omnibus (GEO) database (http://www.ncbi.nlm.nih.gov/geo/), which contains 5,902 single cells from 18 oral squamous cell carcinoma (OSCC) patients based on Illumina NextSeq 500. The bulk RNA-seq profiles of OSCC (32 normal samples and 329 OSCC tumor samples) and their corresponding clinical information are obtained from The Cancer Genome Atlas (TCGA) database (https://portal.gdc.cancer.gov/). Tumor Mutational Burden (TMB) data are downloaded from TCGA database. R software and Perl are used for data preprocessing.

### Processing of the OSCC scRNA-seq Data

The quality control, statistical analysis, and exploration of the scRNA-seq data are all completed by Seurat package in R software, and 5,561 cells are included in the analysis based on the following quality control standards: (1) genes detected in <3 cells and cells with <200 total detected genes cells were excluded; (2) 2,000 < nCount_RNA < 20,000 and nFeature_RNA > 2,000 cells were included in the analysis. Then, the gene expression of the filtered cells was normalized by linear regression model, and the significant available dimensions were determined by principal component analysis (PCA). The t-distributed stochastic neighbor embedding (tSNE) was used for dimensionality reduction and carrying out cluster classification analysis. The singleR package was used for cell clusters annotation according to their characteristic marker genes.

### Analysis of Differentially Expressed Genes and Functional Enrichment

Findmarker function provided by Seurat and DEsingle package was used to recognize differentially expressed genes (DEGs). p-Value < 0.05 and | log2 fold change (FC) | > 0.5 were used as the threshold. Gene Ontology (GO) (18), Kyoto Encyclopedia of Genes and Genomes (KEGG) (19), and Gene Set Enrichment Analysis (GSEA) were used for gene function enrichment analysis (20).

### Cell–Cell Communication Analysis With CellPhoneDB2

CellPhoneDB2 is a Python-based computational analysis tool that can analyze cell–cell communication at the molecular level (21, 22). We used CellPhoneDB2 to analyze the communication patterns between different cells with a p < 0.05.

### Identification of OSCC Cells and Pseudotime Trajectory Analysis

OSCC cells were identified by using InferCNV package (23) to detect the level of copy number variations (CNVs) in epithelial cells (*EPCAM*+). Epithelial cells with high levels of CNVs are defined as malignant epithelial cells, namely, OSCC cells. In order to study the development trajectory of OSCC cells, Monocle2 was used for pseudotime analysis (24). DEGs of pseudotime was analyzed through GeneTest function in Monocle2 with a p < 0.05.

### Correlation Analysis of Immune Characteristics and Tumor Immunogenicity

ESTIMATE algorithm was used to evaluate the purity of OSCC samples, and the results are presented in the form of stromal score, immune score, and ESTIMATE score (25). The data from xCell database (26) and single-sample Gene Set Enrichment Analysis (ssGSEA) were used to explore the association between the expression of *CCDC43* and immune cells infiltration. The xCell database is based on a new method of gene signature to calculate immune cells infiltration. ssGSEA standardizes the gene expression value of a given sample by rank and then calculates the enrichment score by using the empirical cumulative distribution function to obtain the immune infiltration level of each sample. Immune-related gene sets for ssGSEA are from Charoentong et al. (27). The gene lists used to analyze the correlation with CCDC43 are from Thorsson et al. (28). DNA damage repair (DDR)-related gene sets are from Zhang et al. (29).

### Statistical Analysis

All statistical analysis and graph drawing were performed with R (version 4.0.2) and GraphPad Prism (version 8.0). Mann–Whitney U-test was used to compare the difference within two groups, and Kruskal–Wallis test was used to compare the difference between multiple groups. Log-rank test was applied in survival analysis. Pearson or Spearman rank correlation test was used for correlation analysis. Chi-square test is used for sample rate analysis. p-Value < 0.05 is considered to be statistically significant and represented by *, p < 0.01 by **, p < 0.001 by ***, and p < 0.0001 by ****.

## Results

### Identification of 18 Cell Clusters in OSCC Using scRNA-seq Data Reveals High Cell Heterogeneity

Following the quality control standard, 5,902 cells were finally included in our analysis (**Supplementary Figure S1A**). Then, 20 principal components (PCs) were identified by performing principal component analysis (PCA) (**Supplementary Figure S1B**). **Supplementary Figure S1C** showed 2,000 highly variable genes, and the top 10 significantly correlated genes are displayed as dot plots. Subsequently, the t-distributed stochastic neighbor embedding (tSNE) algorithm was applied to cluster the data after PCA dimensionality reduction, and 18 clusters were generated correspondingly. Based on characteristic marker genes (**Supplementary Table S1**), 18 clusters were annotated into 9 different types of cells (**Supplementary Figure S1D**). The heatmap exhibited the top 5 DEGs in each type of cell (**Supplementary Figure S1E**).

### The Percentage of Alternately Activated Macrophages (M2) Decreases at the Site of Lymph Node Metastasis While Cytotoxic T Cells Increase

**Figure 1:**
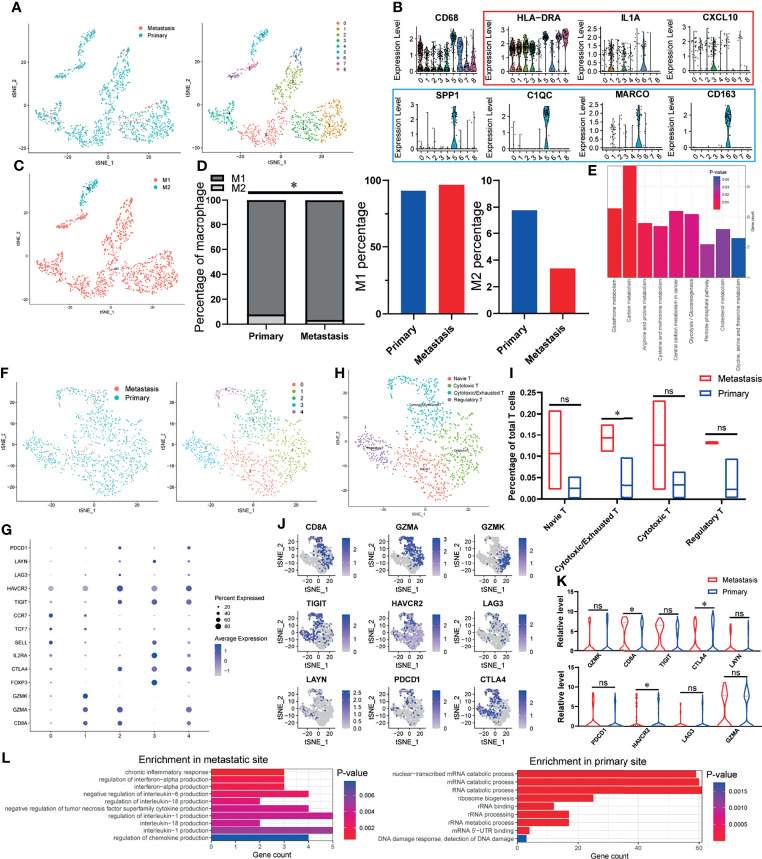
The TIME of OSCC at the site of lymph node metastasis is enriched with classical activated macrophages (M1 macrophages) and cytotoxic T cells. **(A)** tSNE algorithm is used to divide macrophages into eight clusters. **(B)** Violin plot shows the expression of canonical M1 macrophages (red) and M2 macrophages (blue) marker genes in all the macrophages (*CD68*-expressing cells). **(C)** The eight clusters of macrophages are defined as M1 and M2 macrophages. **(D)** Specific content of M1 and M2 macrophages at primary and metastatic sites. **(E)** DEGs-enriched KEGG pathways macrophages between primary and metastatic sites. **(F)** tSNE algorithm is used to divide T cells into five clusters. **(G)** The bubble chart shows the marker genes of cytotoxic T cells (*CD8A*, *GZMA*, *GZMK*), regulatory T cells (*FOXP3*, *IL2RA*, *CTLA4*), naive T cells (*SELL*, *TCF7*, *CCR7*), and exhausted T cells (*TIGIT*, *HAVCR2*, *LAG3*, *LAYN*, *PDCD1*, *CTLA4*). **(H)** All fi e clusters of T cells are annotated according to the composition of the marker genes. **(I)** The proportions of T-cell subtypes in metastatic and primary sites. **(J, K)** Expression of cytotoxic and exhausted T cells’ marker genes at metastatic and primary sites. **(L)** Enriched GO functions of upregulated genes in T cells at metastatic and primary sites. *P < 0.05, ns means no significance.

Since the immune cells in TIME play an essential role in the occurrence and development of tumor, we first explored the characteristics of immune cells infiltration in OSCC samples. As shown in **Figure 1A**, we divided all the macrophages into eight heterogeneous subclusters and grouped all heterogeneous subclusters into classical activated macrophages (M1 macrophages) and alternately activated macrophages (M2 macrophages) types according to characteristic marker genes (**Figures 1B, C** **)**. Through the analysis of the specific content of macrophages, increased M1 and decreased M2 were found in the TIME of lymph node metastasis site compared with primary site (**Figure 1D**). KEGG enrichment analysis found that DEGs of macrophages in primary and metastatic sites were significantly enriched in many metabolic-related pathways, such as cholesterol metabolism, glutathione metabolism, and carbon metabolism (**Figure 1E**). It is reported that the outflow of cholesterol from macrophages membrane can reverse the tumor-promoting function of tumor-associated macrophages (TAMs) (30), which suggests that the difference in cholesterol metabolism between primary and metastatic sites of lymph nodes may lead to different effect functions of macrophages on OSCC cells. Similar to macrophages, we subdivide T cells and finally get five different heterogeneous subclusters (**Figure 1F**). According to specific marker genes (**Figure 1G**), we identified four different types of cell populations, including naive T cells, cytotoxic T cells, regulatory T cells, and an intermediate state between cytotoxic T cells and exhausted T cells (cytotoxic/exhausted T cells) (**Figure 1H**). The analysis of cell content of each subgroup showed that cytotoxic/exhausted T cells in the lymph node metastasis site is significantly higher than that in the primary site (**Figure 1I**). In order to explore the tendency of cytotoxic/exhausted T cells between cytotoxic and exhausted T cells, we specifically studied the expression of cytotoxic and exhausted T cells marker genes in primary and metastatic sites (**Figure 1J**). The results showed that the marker gene *CD8A* of cytotoxic T cells increases significantly at the site of lymph node metastasis, while the marker genes *CTLA4* and *HAVCR2* of exhausted T cells decrease significantly (**Figure 1K**), which suggests that the T cells in the intermediate state is more inclined to the characteristics of cytotoxic T cells at the metastatic site, while the primary site is more similar to exhausted T cells. In order to explore the possible mechanism of this difference, we carried out gene function enrichment analysis of DEGs for T cells between primary and metastatic sites. The results revealed that T cells at the metastatic site are closely related to the production of cytokines and inflammation (interleukin-1 production, interferon-alpha production, etc.), while T cells at the primary site are significantly associated with catabolism progress of nucleic acid (**Figure 1L**).

### Primary Site and OSCC Cells With Lymph Node Metastasis Have Distinct Expression Signatures

**Figure 2:**
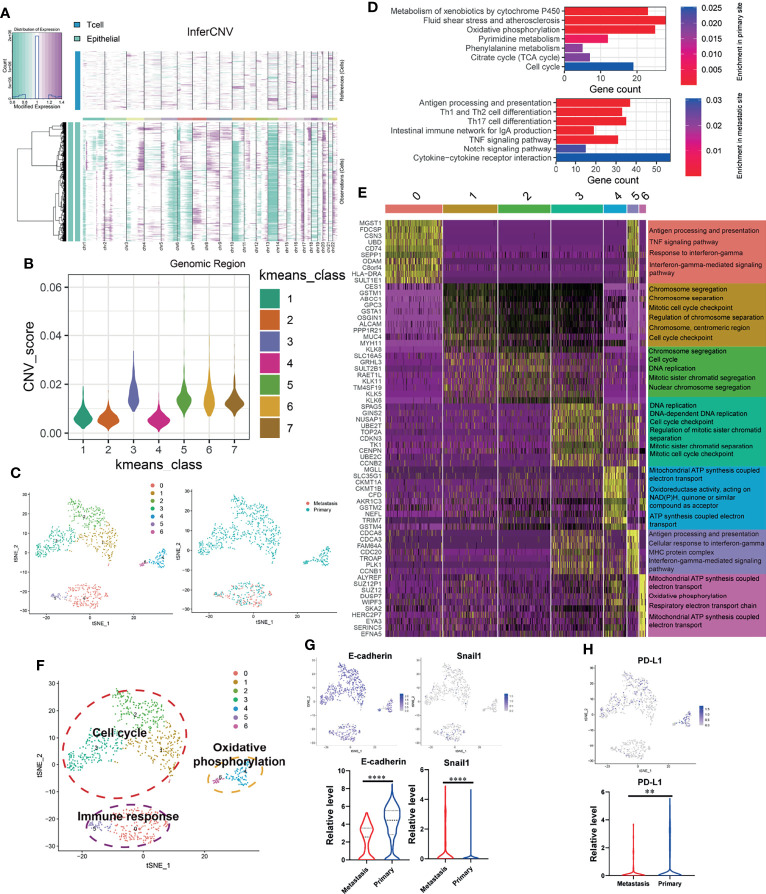
OSCC tumor cells at primary and lymph node metastasis have significant heterogeneous gene expression characteristics. **(A)** Copy number variation (CNV) in epithelial cell; the T cells are used as a reference. Purple represents the high level of CNV, and green represents the low level. **(B)** Kmeans algorithm clustering all epithelial cells into seven clusters according to the level of CNV. **(C)** Using tSNE algorithm to reduce the dimensionality of epithelial cells with high CNV level (OSCC cells, Kmeans 3,5,6,7) into seven clusters. **(D)** Enriched KEGG pathways of upregulated genes in OSCC cells at metastatic and primary sites. **(E)** Heatmap showing the expression of DEGs in each OSCC cluster and their enriched GO functions. Clusters 1–3 are significantly enriched in cell-cycle-related biological functions. Clusters 0 and 5 are significantly enriched in biological functions related to immune response. Clusters 4 and 6 are significantly enriched in the biological functions related to electron transport in respiratory chain and oxidative phosphorylation. **(F)** tSNE plot of all OSCC cells shows seven distinct tumor cell clusters. They can be grouped into cell cycle, immune response, and oxidative phosphorylation based on the functional enrichment analysis of their DEGs. **(G, H)** The expression of *E-cadherin*, *Snail1*, and *PD-L1* in OSCC cells at primary and metastatic sites. **P < 0.01, ****P < 0.0001.

Since significant intratumor heterogeneity exists in OSCC tumor cells, the revelation of its heterogeneity will help to strengthen the understanding of its development process. First of all, we used inferCNV package to calculate the CNV of all epithelial cells (*EPCAM*+). By calculating the CNV level (**Figure 2A**), cells with abnormally high levels of CNV (Kmeans class 3, 5, 6, 7) were identified as malignant epithelial cells (OSCC cells) for follow-up analysis (**Figure 2B**). Then, we clustered the identified OSCC cells into seven different subgroups (**Figure 2C**). **Figure 2D** shows the KEGG enrichment results of upregulated genes of OSCC cells at primary and metastatic sites. According to the functional enrichment analysis of DEGs (**Figure 2E**), OSCC cells were further divided into three functional clusters, which included cell cycle, oxidative phosphorylation, and immune response (**Figure 2F**). Interestingly, OSCC cells at the lymph node metastasis site were all concentrated in the cluster of immune response, which indicates that they may have stronger immunogenicity. By analyzing the expression of *E-cadherin*, *Snail1* (**Figure 2G**), and *PD-L1* (**Figure 2H**), we found that type III epithelial mesenchymal transition (EMT) (31) may play an essential role in the metastasis of OSCC cells, while the depletion of cytotoxic T cells mediated by *PD-L1* expressed on OSCC cells may not be the core factor in this process. In the meantime, the expression of *PD-L1* in the lymph node metastasis site is significantly downregulated, which may also explain why the content of cytotoxic T cells of metastasis is higher than that in the primary site.

### Inflammatory Cancer-Associated Fibroblasts Can Affect M2 Macrophages Infiltration *via* CXCL12

**Figure 3:**
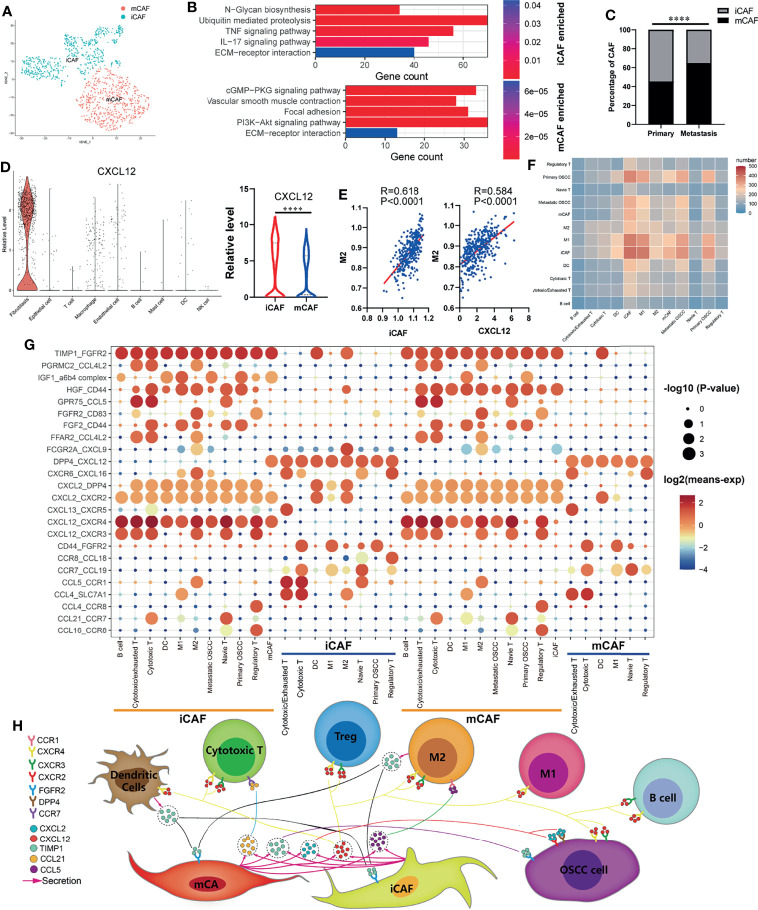
Cell–cell communication network in OSCC TIME. **(A)** Cancer-associated fibroblasts (CAFs) of OSCC are annotated as inflammatory cancer-associated fibroblasts (iCAFs) and myo-cancer-associated fibroblasts (mCAFs) according to their characteristic marker genes. **(B)** Enriched KEGG pathways of upregulated genes in iCAFs and mCAFs. **(C)** Percentage content of MCAF and ICAF in metastatic and primary sites. **(D)** Expression levels of *CXCL12* in various types of cells. **(E)** Correlation analysis between iCAFs content, *CXCL12* expression, and M2 macrophages infiltration level. **(F)** Heatmap showing the number of potential ligand–receptor pairs between all types of cells predicted by CellphoneDB2. **(G)** Using bubble plots to exhibit ligand–receptor pairs of growth factors and cytokines between iCAFs/mCAFs and other cell groups. **(H)** Predicted cell–cell communication network centered on iCAFs and mCAFs. ****P < 0.0001.

The cancer-associated fibroblasts (CAFs) in OSCC were divided into inflammatory cancer-associated fibroblasts (iCAFs) and myo-cancer-associated fibroblasts (mCAFs) (**Figure 3A**) according to their characteristic marker genes (**Supplementary Figure S2A**). KEGG enrichment analysis was used to explore the different functions of iCAFs and mCAFs. As shown in **Figure 3B**, iCAFs are closely associated with tumor necrosis factor (TNF) signaling pathway, *IL-17* signaling pathway, and extracellular matrix (ECM)-receptor interaction. Similarly, ECM-receptor interaction, vascular smooth muscle contraction, and focal adhesion are enriched in mCAFs. By analyzing the specific content of CAFs, we found that iCAFs significantly decreased at the site of lymph node metastasis, while mCAFs significantly increased (**Figure 3C**). In the process of exploring the expression of cytokines in all cells, iCAFs were found to be the main source of *CXCL12* (**Figure 3D**), and the infiltration of M2 macrophages were both positively correlated with iCAFs and *CXCL12* (**Figure 3E**). Basing on CellphoneDB2, we constructed a communication network among all the cell components in TIME (**Figure 3F**). Interaction pairs of growth factor and chemokine are displayed in **Figure 3G**. Previous studies on melanoma found that *CXCL12* can promote the infiltration of TAM by acting on *CXCR4 (* 32). Consistent with previous literature reports, in our cellular communication network, it is found that iCAFs can interact with *CXCR4* expressed in M2 macrophages *via CXCL12*. Therefore, combined with previous research results, we suggest that iCAFs can recruit M2 macrophages by secreting *CXCL12*. Moreover, interaction pairs of *TIMP1*-*CXCR2*/*DPP4*, *CXCL12*-*CXCR3*/*CXCR4*, and *TIMP1*/*FGFR2* play an essential role in the communication between OSCC cells and CAFs. Finally, based on the above analysis results, we plotted a pattern diagram including various cell interactions (**Figure 3H**).

### Pseudotime Trajectory Analysis Indicates That CCDC43 Is Upregulated in More Advanced OSCC Cells and Significantly Associated With OSCC Patients’ Prognosis

**Figure 4:**
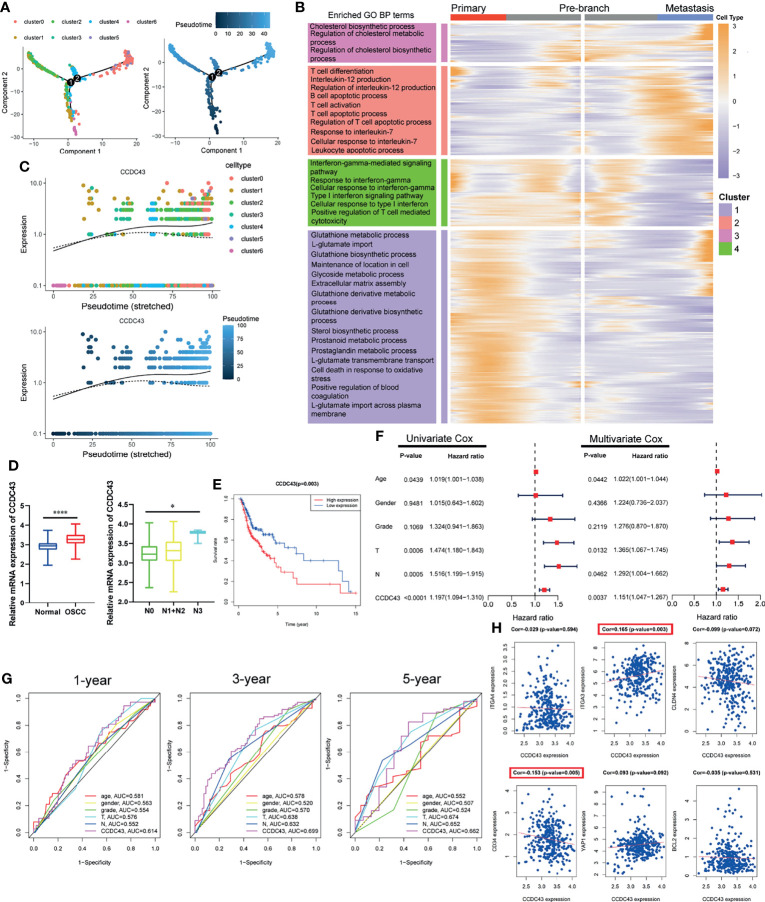
Pseudotime trajectory and clinical prognosis analysis revealed the important clinical prognostic significance of *CCDC43*. **(A)** Pseudo-trajectory and cell source transition of OSCC cells. **(B)** Heatmap exhibits the gene expression dynamics of OSCC cells group. Genes (rows) are clustered, and cells (columns) are ordered according to the pseudotime development. **(C)** Jitter plots showing the expression level of *CCDC43* changing with pseudotime. **(D)** TCGA data set showing that *CCDC43* expression is significantly increased in OSCC samples (compared with normal samples) and lymph node metastasis samples (compared with primary samples). **(E)** Survival analysis showing that overexpression of *CCDC43* significantly worsens the survival outcome of OSCC patients. **(F)** Univariate and multivariate Cox hazards analysis of *CCDC43*, age, gender, grade, T stage, and N stage. **(G)** Time-dependent ROC curve showing the area under curve (AUC) of *CCDC43*, age, gender, grade, T stage, and N stage for 1, 3, and 5 years. **(H)** Correlation analysis of *CCDC43* and classic OSCC markers (*ITGA4*, *ITGA3*, *CLDN4*, *CD34*, *YAP1*, *BCL2*). *P < 0.05, ****P < 0.0001.

In order to further explore the status of OSCC cells at primary and lymph node metastatic sites, we simulate the motion trajectories of OSCC cells from two sources and established the tree-like structure of the whole pedigree differentiation trajectory (**Figure 4A**). In the structure of the tree-like structure that we constructed, the cells at the base are almost entirely composed of OSCC cells at the primary site, while the metastatic OSCC cells are all concentrated in the more advanced state. Then, we carried out DEGs analysis according to the expression in different time states, and it was divided into four clusters according to the expression pattern, which is displayed in the form of ranched heatmap. Gene Ontology (GO) enrichment analysis showed the characteristic functions of different clusters (**Figure 4B**). In the following analysis, we performed Cox survival analysis of all pseudotime-associated DEGs using the clinical data from TCGA database (**Supplementary Table S2**), and the result indicates that *CCDC43* is the most significant gene related to the survival of OSCC patients (**Supplementary Table S3**). **Figure 4C** shows that the expression of *CCDC43* in more advanced OSCC cells has an upregulation trend. Then, we found that the expression of *CCDC43* is significantly upregulated in OSCC samples compared with that in the normal samples and closely associated with lymph node metastasis of OSSC (**Figure 4D**). Other than this, it was found that high expression of *CCDC43* can worsen the survival status of OSCC patients (**Figure 4E**). In order to further evaluate the prognostic significance of *CCDC43*, we combined *CCDC43* with the clinical indicators (age, gender, grade, T stage, and N stage) for univariate and multivariate Cox hazard regression analysis. As a result, compared with the above clinical indicators, *CCDC43* exhibits its superiority and is an independent prognostic factor (**Figure 4F**). Then, we plotted the time-dependent ROC curves of OSCC patients (1, 3, and 5 years), which also confirmed the essential prognostic value of *CCDC43* (**Figure 4G**). Subsequently, we performed coexpression analysis of *CCDC43* and classical OSCC markers (33), and we found that *CCDC43* is positively correlated with *ITGA3*, while it is negatively correlated with *CD34* (**Figure 4H**).

### High Expression of CCDC43 Is Related to Impaired Antitumor Immunity and Enhances DNA Damage Repair Function

**Figure 5:**
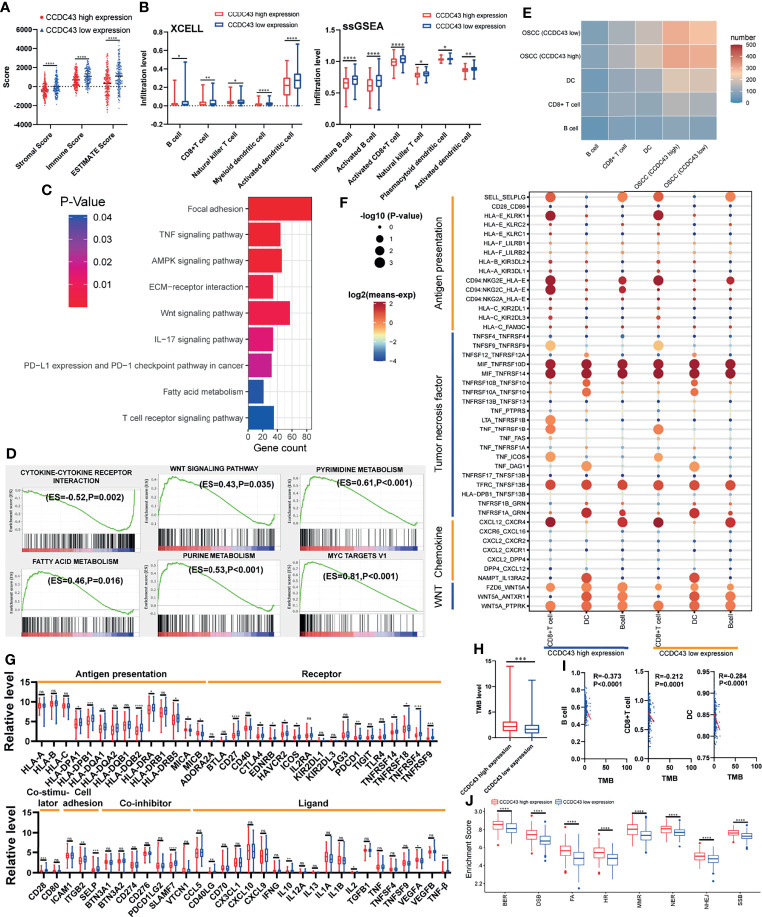
Correlation between *CCDC43* expression and immune characteristics and tumor immunogenicity. **(A)** ESTIMATE algorithm calculating the immune score, stromal score, and ESTIMATE score of the group with high and low expression of *CCDC43*. **(B)** Using XCELL database and ssGSEA algorithm to analyze the correlation between *CCDC43* and immune cells infiltration. **(C, D)** Using KEGG and GSEA to annotate the function of *CCDC43*. **(E)** Heatmap showing the number of potential ligand–receptor pairs between OSCC cells with high-/low-expression *CCDC43* and B cell, CD8+ T cell, and dendritic cell (DC). **(F)** Using bubble plots to exhibit ligand–receptor pairs of antigen-presentation-associated molecules, tumor necrosis factor, chemokine, and WNT signaling pathway-associated molecules between high/low *CCDC43* expression OSCC cells and immune cells (CD8+ T cell, DC, and B cell). **(G)** Box diagram depicting the expression level of immune-related genes between *CCDC43* high- and low-expression group. Red represents *CCDC43* high-expression group, and blue represents *CCDC43* low-expression group. **(H)** Analysis of the association between *CCDC43* expression and tumor mutation load (TMB). **(I)** Correlation analysis of TMB and the infiltration level of B cell, *CD8*+ T cell, and DC. **(J)** The enrichment of DNA damage repair-related gene set between *CCDC43* high- and low-expression group. *P < 0.05, **P < 0.01, ***P < 0.001, ****P < 0.0001, ns means no significance.

**Figure 6:**
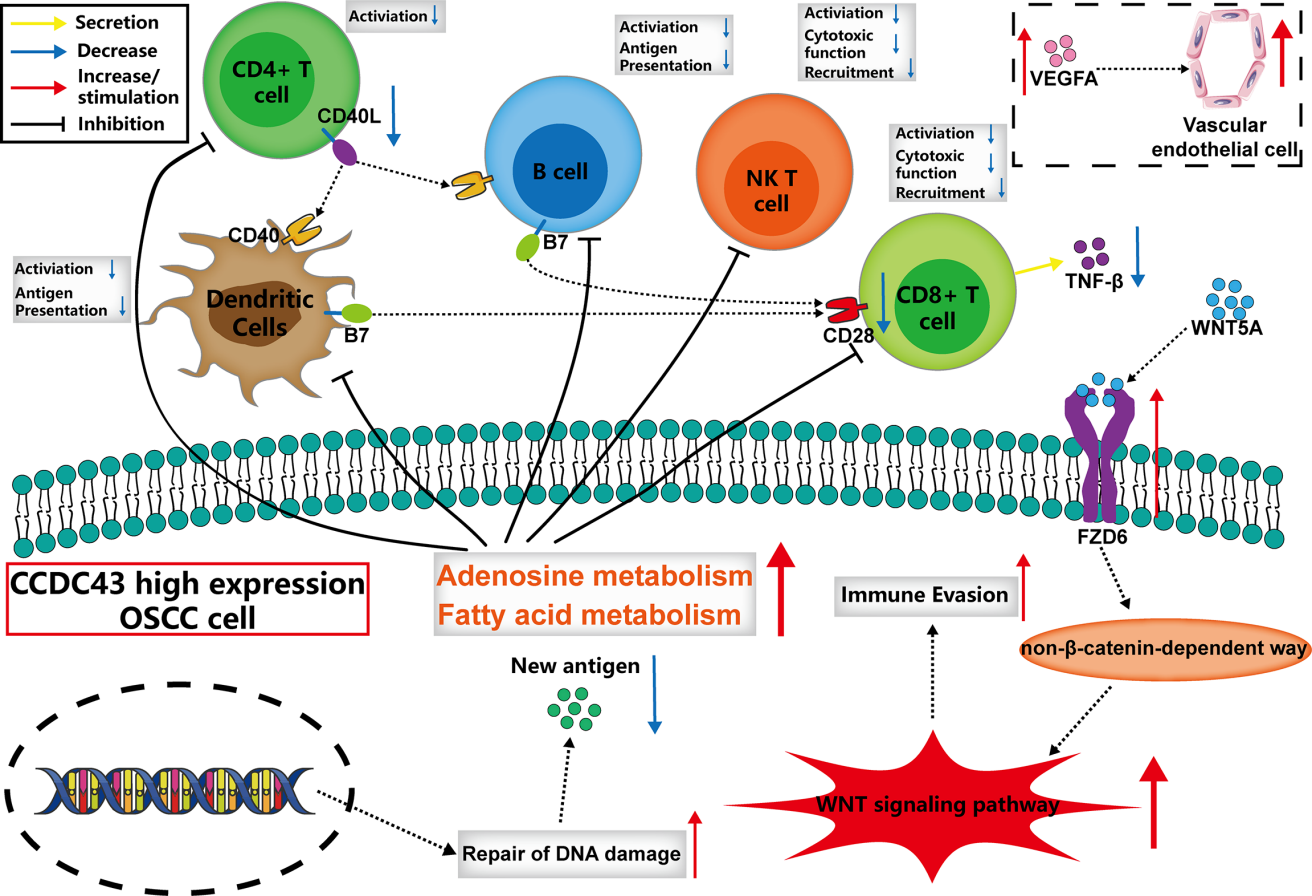
The possible mechanism of the function of *CCDC43* expression on tumor immunity.

To explore the potential function of *CCDC43* in TIME, the ESTIMATE algorithm was first used to evaluate the general condition of stromal cells and immune cells. The results showed that the high expression of *CCDC43* leads to a significant decrease in the infiltration level of both immune cells and stromal cells (**Figure 5A**). The analysis of xCell database and ssGSEA algorithm revealed that in the samples with high expression of *CCDC43*, the infiltration levels of B cells, *CD*8+ T cells, natural killer T cells, and dendritic cells decreases significantly (**Figure 5B**). This finding indicated that immune-activated cells are significantly inhibited in *CCDC43* high-expression group. To study the possible mechanism of the effect of *CCDC43* on immune cells infiltration, KEGG enrichment analysis and GSEA were first conducted. **Figure 5C** shows immune-associated pathways related to the differential expression of *CCDC43*. GSEA showed that immunosuppression-related pathways (WNT signaling pathway, fatty acid metabolism, etc.) are significantly upregulated in the *CCDC43* high-expression group, while immune activation-related pathways (cytokine–cytokine receptor interaction, etc.) are concentrated in the low-expression group (**Figure 5D**). Then, cell–cell communication was carried out to further explore the communication patterns between OSCC cells with different *CCDC43* expression and CD8+ T cells, B cells, and dendritic cells. **Figure 5E** shows that OSCC cells with high *CCDC43* expression has more communication with the above three kinds of cells. Considering the results of gene functional enrichment analysis, we further displayed the interaction pairs in antigen presentation, TNF, chemokine, and WNT signaling pathway (**Figure 5F**). It is reported that abnormal activation of WNT signaling pathway can significantly reduce the degree of T-cell infiltration and increase the unresponsiveness of immunotherapy (34, 35). Interestingly, our study finds that the interaction of *WNT5A*-*FZD6* is significantly enhanced in *CCDC43* high-expression OSCC cells compared with that in the low-expression group, which suggests that *CCDC43* may be involved in the process of T-cells depletion mediated by WNT signaling pathway. In the process of exploring the expression of tumor-immunity-related genes, it is found in the group with high expression of *CCDC43* that the expression of antigen-presentation-related molecules, immune-related ligands, receptors, and co-suppressor molecules are generally downregulated (**Figure 5G**). We also found that the high expression of *CCDC43* is closely related to the higher level of TMB (**Figure 5H**), while there is a significant negative correlation between TMB and the infiltration level of B cells, CD8+ T cells, and dendritic cells (**Figure 5I**), which suggests that high levels of TMB cannot be used as an effective predictor (36) in other cancers as previously reported. In addition, DNA damage repair (DDR) pathways were found to be significantly upregulated in the *CCDC43* overexpression group (**Figure 5J**). Based on the above analysis results, we hypothesized the possible mechanism by which CCDC43 functions in OSCC (**Figure 6**).

## Discussion

In recent years, exploring the basic role of TIME in regulating the development of HNSCC has been the focus of various studies. More and more attention has been paid to the study of the function of the main cellular components of TIME and how these cells interact through the complex communication network through the expression of growth factors, cytokines, and chemokines and their counterparts with malignant cells (37). The interaction between tumor cells and TIME is an essential factor for tumor progression and metastases, and its antitumor or antitumor-promoting properties have been widely studied for the potential immunotherapy of HNSCC (38, 39). For instance, it has been reported that terminal depletion of tumor-antigen-specific T lymphocytes will lead to ineffective blocking of immune checkpoints. In addition, M2 macrophages and Treg can also induce T-cell depletion by expressing immunosuppressive cytokines that promote immunosuppression of TME (40), which may lead to tumor recurrence after the termination of acquired drug resistance and immune checkpoint blocking therapy in HNSCC (41). Therefore, an in-depth study of the heterogeneity of HNSCC’s TIME will help to develop new immunotherapy applications to improve the prognosis of patients. However, as an important type of HNSCC, few previous studies have put the single-cell transcriptome of OSCC cells and their TIME together to study. In the present study, based on the scRNA-seq data of OSCC samples, we comprehensively and carefully analyzed the tumor cells and their TIME components at the primary and metastatic sites and found that both of OSCC cells and TIME components had distinct heterogeneity, of which the meaning is helpful to further study the mechanism of OSCC cells metastasis and guide the clinical treatment of OSCC patients.

Cytotoxic T cells are the most important cells in the body to exert antitumor immunity, which are produced by the activation of *CD8*+ T cells in a process called tumor immune circulation. In this process, the increased immunogenicity of tumor cells contributes to its activation, while CAF, M2 macrophages, and regulatory T cells (Tregs) inhibit the activation of cytotoxic T cells (42). The enhancement of some metabolic processes(cholesterol metabolism, adenosine metabolism, etc.) (43) and the overexpression of *PD-L1* on tumor cells can increase the depletion of cytotoxic T cells. In OSCC, we find that the content of cytotoxic T cells at the lymph node metastatic site is significantly higher than that at the primary site, which may be due to the decrease in M2 macrophages at the metastatic site, the weakening of cytotoxic T cells depletion mediated by *PD-L1* expressed in OSCC cells, and the increased immunogenicity of tumor cells. CAFs are an inhibitory intermediate in TIME, which can kill cytotoxic T cells in an antigen-dependent manner and is associated with poor prognosis (44). *CXCL12* is an important chemokine, and high levels of it in TIME can recruit highly tumorigenic tumor cells and promote their proliferation, angiogenesis, and metastasis (45). We found that in OSCC, *CXCL12* is mainly secreted by CAFs, especially iCAFs. According to literature reports, the receptors of *CXCL12* mainly include *CXCR3*, *CXCR4*, and *CXCR7 (* 46), and *CXCR4* is widely expressed on various immune cells in OSCC, which suggest that *CXCL12* secreted by iCAFs is a key factor determining the immune infiltration of OSCC. Correspondingly, we find that iCAFs may promote M2 macrophages infiltration by secreting *CXCL12*. What is interesting is that the content of iCAFs at the site of tumor metastasis decreases significantly, so it is suggested that the decrease in the content of M2 macrophages is partly because of iCAFs, which is worthy of further exploration. Other than this, basing on some bioinformatics methods, we constructed a communication network among all cells in TIME, and iCAFs are found to communicate most with no matter OSCC cells or other immune cells in this network, which indicates that iCAFs play an irreplaceable role in the occurrence and development of tumor cells and the function of various immune cells. Similarly, iCAFs are found to affect the process of epithelial–mesenchymal transformation in colon cancer cells by secreting *IL-6* in previous studies (47).

*CCDC43* is a member of the coiled-coil domain-containing (CCDC) family, and CCDC is considered to be involved in the proliferation, invasion, and metastasis of malignant tumor cells (48, 49). In previous studies, high expression of *CCDC43* was found to promote metastasis of gastric and colon cancer (50, 51), but so far, there is no research on *CCDC43* in OSCC. In our study, we found that the expression of *CCDC43* is significantly increased in OSCC samples and is closely related to lymph node metastasis and patients’ prognosis. By analyzing the correlation between *CCDC43* and TIME, it is found that high expression of *CCDC43* can significantly inhibit antitumor immunity and increase immune escape of OSCC cells. There may be various reasons for the above functions of *CCDC43*, including the inhibition for the infiltration of essential antitumor immune cells, the decrease in autoimmunogenicity of OSCC cells (52), the increased level of substances that inhibit the activity of immune cells (adenosine, fatty acid, etc.) (43), abnormal activation of WNT signaling pathway (34, 35), and so on. Based on the above analysis results, we described the possible mechanism by which *CCDC43* functions in TIME. That is, *CCDC43* can affect the recognition and immune response of immune cells with OSCC cells in TIME by affecting the process of lipid metabolism and DNA damage repair progress in OSCC cells. At the same time, *CCDC43* can activate the non-β-catenin-dependent WNT signaling pathway by upregulating the expression of *FZD6* on the membrane of OSCC cells (53) and then increase the immune escape ability of OSCC cells and promote its metastasis. Therefore, we suggest that *CCDC43*-associated function mechanism in OSCC is worthy of using concrete experimental technology to explore further.

Early detection, early diagnosis, and early intervention are the most effective ways to treat OSCC. However, in patients with OSCC, it is usually difficult to diagnose at the early stage. Therefore, in order to more systematically reveal the process and mechanism of the occurrence and development of OSCC, the integration of multi-omics methods came into being and promoted the transformation of OSCC research paradigm from single-parameter model to multiparameter system model. For example, Kagohara et al. revealed the heterogeneity and early changes in pathways associated with cetuximab resistance in HNSCC-sensitive cell lines through integrated single-cell and bulk gene expression and ATAC-seq (54). Chen et al. used single-cell transcriptome combined with related experiments to reveal the intratumoral landscape of infiltrating T-cell subsets in oral squamous cell carcinoma, which provided a valuable insight for understanding the functional status and heterogeneity of T-cell populations in OSCC (55). In the present study, we also integrate multiple omics data to explore the possible mechanism of OSCC metastasis. The integration of multi-omics methods is expected to clarify the mechanism of the occurrence and development of OSCC, find biomarkers with the function of diagnosis and prognosis, explore new treatment targets, and finally realize the prediction, prevention, and individualized treatment of OSCC.

In summary, our work provides a valuable resource for understanding the heterogeneity of OSCC cells and corresponding TIME between primary and metastatic sites of OSCC patients. We discover the important value of *CCDC43* in clinical prognosis and tumor immunity and reveal the possible mechanism by which it mediates tumor cell interactions with other cells in TIME, which indicates that *CCDC43* is expected to become an important therapeutic target for OSCC.

## Data Availability Statement

The datasets presented in this study can be found in online repositories. The names of the repository/repositories and accession number(s) can be found below: https://www.ncbi.nlm.nih.gov/geo/, GSE103322, and https://portal.gdc.cancer.gov/, OSCC samples.

## Author Contributions

MY conceived and supervised the article. ZW collected and analyzed the data, wrote the manuscript, and generated the figures. HZ and YZ generated the tables. FL and XS revised the manuscript. All authors contributed to the article and approved the submitted version.

## Funding

This work was supported by grants from the National Nature Science Foundation of China (31160233), the Science and Technology Foundation of Jiangxi Province (20142BAB204013), and Graduate Student Innovation Special Foundation of Jiangxi Province (YC2018-S087).

## Conflict of Interest

The authors declare that the research was conducted in the absence of any commercial or financial relationships that could be construed as a potential conflict of interest.

## Publisher’s Note

All claims expressed in this article are solely those of the authors and do not necessarily represent those of their affiliated organizations, or those of the publisher, the editors and the reviewers. Any product that may be evaluated in this article, or claim that may be made by its manufacturer, is not guaranteed or endorsed by the publisher.
