# Supplementary material for: Single-Cell Profiling Reveals Heterogeneity of Primary and Lymph Node Metastatic Tumors and Immune Cell Populations and Discovers Important Prognostic Significance of CCDC43 in Oral Squamous Cell Carcinoma
Source: Front Immunol. 2022 Mar 24;13:843322. doi: 10.3389/fimmu.2022.843322 (PMC8986980; doi:10.3389/fimmu.2022.843322)
Supplement: Supplementary file 1 [file DataSheet_1.zip › Supplementary data/Supplementary figures.docx]

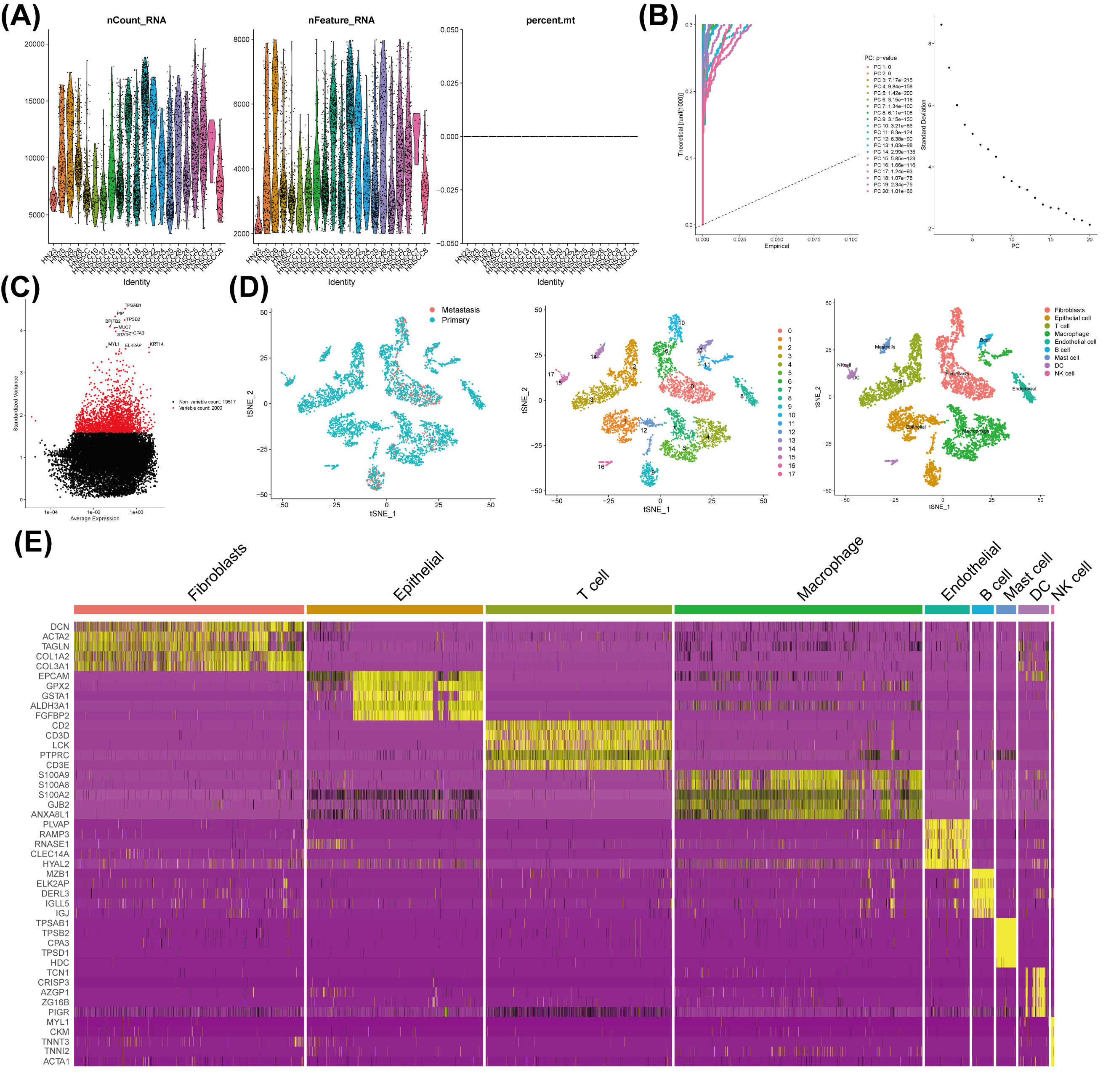


**Figure S1.** Single-cell expression atlas of primary and lymph node metastasis OSCC cells. (A) After quality control of the 5902 cells from OSCC samples, 5561 cells were included in the analysis. (B) PCA identified the 20 PCs with an estimated P value < 0.05. (C) The variance diagram displays 21517 genes throughout all cells from OSCC. The red dots represent highly variable genes, and the black dots represent nonvariable genes. The top 10 most variable genes are marked in the plot. (D) Using tSNE algorithm to reduce the dimensionality of 20 PCs and finally get 18 clusters. According to characteristic maker genes, 18 clusters are defined as fibroblasts, epithelial cell, T cell, Macrophage, endothelial cell, B cell, mast cell, dendritic cell (DC) and natural killer (NK) cell. (E) Heatmap of the top 5 DEGs (p<0.05) in each cell type.


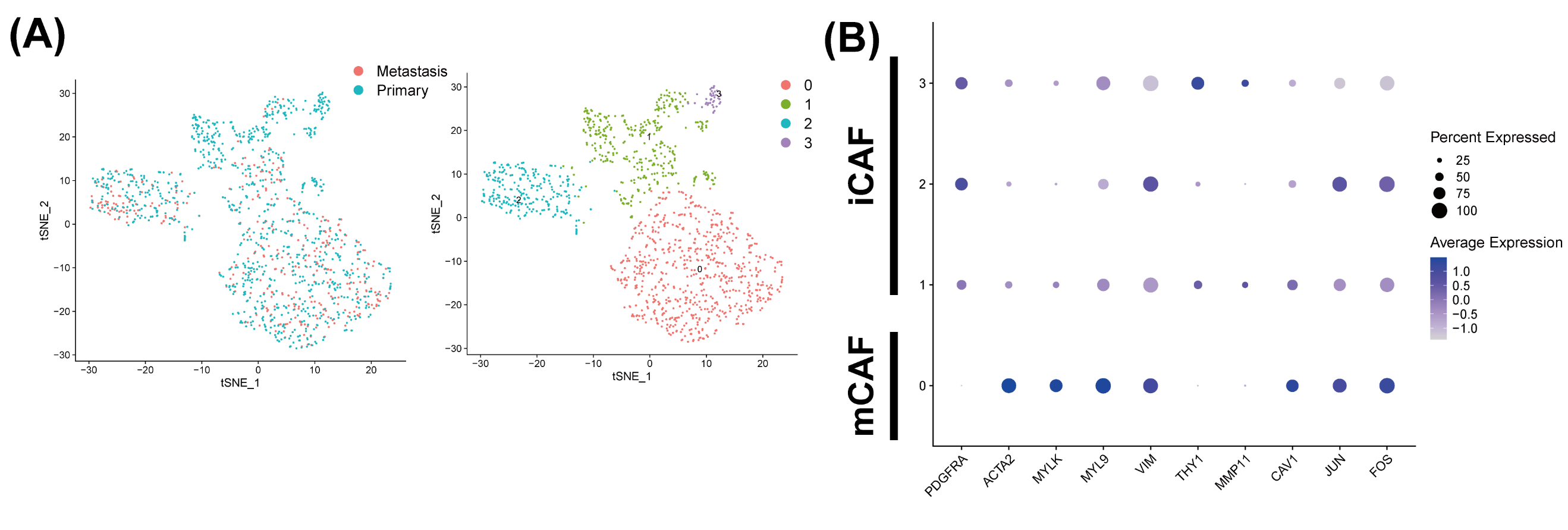


**Figure S2.** Identification of cancer-associated fibroblasts (CAFs) related subsets. (A) CAFs are divided into 4 clusters. (B) Gene makers of iCAFs (inflammatory cancer-associated fibroblasts) and mCAFs (myo-cancer-associated fibroblasts).
