# Supplementary material for: Single-Cell Profiling Reveals Heterogeneity of Primary and Lymph Node Metastatic Tumors and Immune Cell Populations and Discovers Important Prognostic Significance of CCDC43 in Oral Squamous Cell Carcinoma
Source: Front Immunol. 2022 Mar 24;13:843322. doi: 10.3389/fimmu.2022.843322 (PMC8986980; doi:10.3389/fimmu.2022.843322)
Supplement: Supplementary file 1 [file DataSheet_1.zip › Supplementary data/Table S1.docx]

**Table S1. Canonical markers used to annotate each cell type cluster**

| Cells | Maker genes |
| --- | --- |
| Epithelial cell | EPCAM |
| Endothelial cell | PECAM1 |
| Dendritic cell (DC) | CD1A, CD1C, CD207, CCL17 |
| Mast cell | TPSAB1, TPSB2, CPA3, MS4A2, CLU |
| Fibroblasts | COL3A1 |
| B cell | CD79A, CD37, CD79B, IGHG1, IGHA1, IGHM |
| Natural killer cell | GNLY, NKG7, CD160, GZMB, CCL3 |
| Macrophage | CD14, CD163, CD68, CSF1R |
| T cell | CD27, CCR7, CD8A, CD8B, CCR10, CD52, CMTM7,  FOXP3, ZNF683, GZMK, CD4, CD3E |
