# Supplementary material for: Single-Cell Profiling Reveals Heterogeneity of Primary and Lymph Node Metastatic Tumors and Immune Cell Populations and Discovers Important Prognostic Significance of CCDC43 in Oral Squamous Cell Carcinoma
Source: Front Immunol. 2022 Mar 24;13:843322. doi: 10.3389/fimmu.2022.843322 (PMC8986980; doi:10.3389/fimmu.2022.843322)
Supplement: Supplementary file 1 [file DataSheet_1.zip › Supplementary data/Table S2.docx]

**Table S2. Clinical information of OSCC patients from TCGA database**

| Clinical characteristics | Variable | Total (328) | Percentages (%) |
| --- | --- | --- | --- |
| Age | >=65 | 134 | 40.85 |
|  | <65 | 193 | 58.84 |
|  | Unknown | 1 | 0.30 |
| Gender | Male | 227 | 69.21 |
|  | Female | 101 | 30.79 |
|  | Unknown | 0 | 0 |
| Grade | Stage I | 20 | 6.10 |
|  | Stage II | 56 | 17.07 |
|  | Stage III | 59 | 17.99 |
|  | Stage IV | 159 | 48.48 |
|  | Unknown | 34 | 10.37 |
| T | T0 | 1 | 0.30 |
|  | T1 | 32 | 9.76 |
|  | T2 | 103 | 31.40 |
|  | T3 | 65 | 19.82 |
|  | T4 | 101 | 30.79 |
|  | Unknown | 26 | 7.92 |
| M | M0 | 120 | 36.59 |
|  | M1 | 0 | 0 |
|  | Unknown | 208 | 63.41 |
| N | N0 | 118 | 35.98 |
|  | N1 | 49 | 14.94 |
|  | N2 | 104 | 31.71 |
|  | N3 | 3 | 0.91 |
|  | Unknown | 54 | 16.46 |
